# Small-Molecule SB216763-Loaded Microspheres Repair Peripheral Nerve Injury in Small Gap Tubulization

**DOI:** 10.3389/fnins.2019.00489

**Published:** 2019-05-15

**Authors:** Feng Rao, Zhipeng Yuan, Dianying Zhang, Fei Yu, Ming Li, Dongdong Li, Baoguo Jiang, Yongqiang Wen, Peixun Zhang

**Affiliations:** ^1^Department of Orthopedics and Trauma, Peking University People’s Hospital, Beijing, China; ^2^School of Chemistry and Biological Engineering, University of Science and Technology Beijing, Beijing, China

**Keywords:** tubulization, peripheral nerve injury, small-molecule, SB216763, microsphere

## Abstract

Peripheral nerve injury has yet to be fully resolved because of its complicated pathological process. SB216763 is a small molecular compound that can enhance the remyelination of peripheral nerves by inhibiting glycogen synthase kinase-3β (GSK3β). GSK-3β inhibitor stimulates myelin gene expression and restores the myelin structure. Herein, we presented the effect of integrating small gap tubulization with SB216763-loaded microspheres by using a chitosan conduit. *In vitro*, SB216763 could promote neurite growth of dorsal root ganglia. *In vivo* studies showed that SB216763 increased the number of myelinated axons and the thickness of myelin sheaths. Electrophysiological examination and sciatic functional index results also indirectly indicated the role of SB216763 in repairing peripheral nerve injury. SB216763 promoted the recovery of muscle function. Therefore, combining SB216763-loaded PLGA microspheres with conduit small gap tubulization shows potential for applications in repairing peripheral nerve injury.

## Introduction

The treatment of limb paralysis and dysfunction caused by peripheral nerve injury is a medical science problem. Various major problems in peripheral nerve injury and repair have yet to be effectively addressed globally (Noble et al., 1998; Taylor et al., 2008; Asplund et al., 2009).

Given the slow rates of nerve regeneration, tissue adhesion, and muscle atrophy, obtaining satisfactory results for impaired nerve function is difficult. Peripheral nerve damage causes problems for patients and their families, resulting in substantial psychological and economic burden to the society, and their families (Huang et al., 2012). The effective repair of peripheral nerve injury and the repair effect of peripheral nerve injury have become a major public health problem that requires an urgent solution in the current health field.

Peripheral nerve injury is usually repaired by epineurial neurorrhaphy, but the functional recovery is usually unsatisfactory and hindered by many factors, including the misconnections of sensory and motor nerve fibers, scars, and painful neuromas (Huang et al., 2012; Zhang et al., 2015b). Our team takes full advantage of the phenomenon of peripheral nerve chemotaxis regeneration. We proposed a biodegradable chitosan conduit as a substitute to traditional epineurial neurorrhaphy with the small gap tubulization for peripheral nerve injury (Jiang et al., 2006, 2010; Kou et al., 2013; Zhang et al., 2013). The effects of conduit tubulization with a small gap are better than those of epineurial neurorrhaphy, and treatments do not form neuroma and scar tissues.

Small gaps could not only provide a space for nerve-selective regeneration but also offer a local microenvironment for nerve growth (Yu et al., 2009; Zhang et al., 2015a). After peripheral nerve injury, a series of pathophysiological events will occur, thereby leading to Wallerian degeneration in the distal stump. Macrophages and monocytes migrate to nerve stumps to remove myelin and axon debris. New axonal sprouts emanate from Ranvier nodes and undergo remyelination induced by Schwann cells (Gu et al., 2014).

Myelin is essential for the rapid conduction of axonal impulses. Myelination, which is elicited in peripheral nerves by Schwann cells, is a complex and fine-tuned process. The expression of peripheral myelin genes, namely, *myelin protein zero* (*MPZ*) and *peripheral myelin protein 22* (*PMP22*), is strictly regulated (Makoukji et al., 2012).

Glycogen synthase kinase (GSK3) was originally discovered as a glycogen synthase inactive protein kinase. Its related signal transduction pathways have been identified as therapeutic targets for neurodegenerative diseases, such as Alzheimer’s disease (Noble et al., 2005; Maqbool et al., 2016; Kirouac et al., 2017), schizophrenia (Chien et al., 2018), and Parkinson’s disease (Youdim and Arraf, 2004). GSK-3 plays an important role in the regulation of the central and peripheral nervous systems except neurodegenerative diseases.

Emerging evidence indicates the relevant role of GSK3 in peripheral nerve regeneration, suggesting that GSK3 inhibition facilitates axon regeneration *in vivo* (Zhou et al., 2004; Dill et al., 2008; Saijilafu et al., 2013). GSK-3 inhibitors also upregulate the expression of the myelin-associated genes *MPZ* and *PMP22* by activating the Wnt/β-catenin signaling pathway (Su et al., 2007; Tawk et al., 2011). Small molecular compound SB216763, is potent, selective and GSK-3inhibitor for both GSK-3α and GSK-3β. Also, it has no the limitations of short half-life and high cost of neurotrophic factors, such as NGF, which is widely used to repair peripheral nerve injury.

In order to make SB216763 work continuously, we combine the sustained release microsphere system with a biodegradable chitin conduit to repair peripheral nerve injury in small gap tubulization. 12 weeks after operation, histological, and functional data show that the GSK3β inhibitor SB216763 promotes axonal regeneration and remyelination, thereby providing a promising therapeutic approach for peripheral nerve injury. Our data also showed that SB216763 has implications on the treatment of demyelinating diseases.

## Materials and Methods

### Isolation and Culturing of Rat Dorsal Root Ganglion (DRG)

SD rats were used within 12 h after they were born. Their spines were completely cut, and their blood was washed with phosphate buffered saline (PBS). Then, the spine was cut into two halves along the central axis to expose DRGs. DRGs were collected from the intervertebral foramen under a microscope. DMEM/F12 and 10% FBS. The epineurium of the DRGs was removed in a DMEM/F-12 medium supplemented with 10% FBS. Then, the DRGs were planted in a six-well plate containing the DMEM/F12 medium supplemented with B27 and glutamine. In the experimental group, 10 μmol SB216763 was added to the medium.

### Immunofluorescence

After the specimens were cultured for 5 days, mouse anti-Neurofilament 200 was subjected to immunofluorescence staining to evaluate the effect of SB216763 on the DRGs. First, the DRGs were fixed with paraformaldehyde for 30 min. Subsequently, the DRGs were blocked with 10% goat serum for 1 h, and mouse anti-neurofilament 200 (1:800; N0142; Sigma) was applied as the primary antibody of the DRGs at 4°C overnight. Then, goat anti-mouse IgG (Alexa Fluor^®^488; 21 1:200; ab150117; Abcam) secondary antibody was incubated with the DRGs in the dark for 1 h. The three longest axons per quadrant were measured and used to calculate the mean length of each DRG axon by using Image Pro Plus 6.0.

#### Fabrication of Microspheres

SB216763-loaded microspheres and NGF-loaded microspheres were prepared through the modified W/O/W emulsion solvent evaporation method published previously (Wang et al., 2014).

In brief, 0.1 ml of the internal aqueous phase of the SB216763 or NGF solution with 1 mg of BSA as a protective additive was emulsified in 2 ml of 25 mg/ml PLGA dichloromethane solution. The emulsion was sonicated for 30 s in an ice bath to create the primary emulsion. The primary emulsion was added dropwise to 30 ml of the external aqueous phase of 3% (w/v) polyvinyl alcohol solution with continuous stirring at 1500 rpm to obtain multiple emulsions. After 5 min, the resulting emulsion was poured into 300 ml of 0.3% (w/v) PVA solution and stirred for 3 h at room temperature to evaporate the dichloromethane content. Finally, the resulting suspension was centrifuged, and the collected microspheres were washed with deionized water thrice and freeze dried to obtain a free-flowing powder.

#### Evaluation of Microspheres

The morphological characteristics of the PLGA microspheres were examined using a scanning electron microscope (SEM; HITACHI-8010, Japan). Three locations were randomly selected, and the particle size of the microspheres in the field of vision was measured by nanomeasure software. Statistical results were also fitted with Gaussian distribution.

The loading capacity (%) and encapsulation efficiency (%) was quantified: Load capacity (%) = (loaded drug weight/ microsphere weight) × 100%. Encapsulation efficiency (%) = (encapsulated drug weight/total drug weight) × 100%.

SB216763-loaded microspheres and NGF-loaded microspheres were respectively, weighed for 20 mg and placed in centrifuge tube containing 1.5 ml of PBS solution. Release studies were performed at 37°C. To detect the release kinetics of SB216763 and NGF, we determined the total volume of the PBS after centrifugation was performed and replaced it with the same volume of PBS at each sampling time. The cumulative release profiles of SB216763-loaded and NGF-loaded microspheres were evaluated via high-performance liquid chromatography. SB216763 microsphere samples were analyzed on a C18 column with a mobile phase of acetonitrile/water (v/v, 36/64). The flow rate was set to 1 ml/min, and absorbance was monitored with an ultraviolet light detector (UV230II) at 278 nm. All of the samples were assayed in triplicate.

### Fabrication of Chitosan Conduit

Chitosan conduits, patented by Peking University and China Spinning and Weaving Institute, can retain their structure for at least 6 weeks *in vivo* and biodegrade completely (Chinese patent: ZL01136314). Berifly, chitosan powder was diluted by 3% glacial acetic acid. Specific molds 1.5 mm in diameter were used to draw the chitosan solution, pulled out, froze for 1 h in −20°C, then solidified with 5% sodium hydroxide solution for 1 h. Chitosan conduits were pulled out from the mold, dried for 60 min at 50°C, and acetylated for 30 min. Finally, the acetylated chitosan conduits, also called chitin conduits were stored at 4°C after cleaning.

#### Animals and Surgical Procedures

Eight-week-old SD rats were provided by Beijing Vital River Laboratory Animal Technology Co., Ltd. We followed the Laboratory Animal Guideline for the Ethical Review of the Animal Welfare of China (GB/T 35892-2018). All of the experiments were in compliance with the relevant regulations of the Medical Ethics Committee of Peking University People’s Hospital (Approval No. 2102000024). A total of 32 SD rats weighing 200–220 g were randomly divided into four groups: epineurium neurorrhaphy (epineurium group), conduit small gap tubulization-integrated empty microsphere (conduit group), conduit small gap tubulization-integrated SB216763-loaded microsphere (SB216763 group), and conduit small gap tubulization-integrated NGF-loaded microsphere (NGF group) groups. The concentration of the NGF solution was set to 40 ng/ml.

All of the rats were anesthetized with pentobarbital sodium solution (30 mg/kg body weight). The sciatic nerve of the right hind was exposed and cut. The small gap suture group retained a 2 mm gap with a biodegradable conduit, and the microspheres were injected using a microinjector (Figure 3). Then, the muscle and the skin were sutured. After waking, the rats were reared in groups and subjected to automatic feeding of food and drinking water under 12 h of light/dark natural circulation.

#### Transmission Electron Microscopy (TEM) and Morphological Analyses

The regenerated nerve tissues were collected 12 weeks after surgery. Nerve tissue samples were fixed with 2.5% glutaraldehyde, stained with 1% osmium acid, dehydrated with a gradient concentration of acetone, embedded in EPON812 epoxy resin, and cut into 700 nm thick semithin sections and 70 nm thick ultrathin sections. The semithin sections were stained with 1% toluidine blue, and the myelinated nerve fiber density was recorded in eight random images of the rats under light microscopy. The ultrathin sections were dyed with uranyl acetate and lead citrate. The diameter of the myelinated axons and the thickness of the myelin sheath were measured with TEM. The data were analyzed with Image Pro Plus.

#### Electrophysiological Assessment

The rats in each group were subjected to electrophysiological tests 12 weeks after surgery. The sciatic nerve on the injured side were re-exposed under anesthesia. The stimulus intensity and duration parameters of a Medlec synergy electrophysiological system (Oxford Instrument Inc., Abingdon, United Kingdom) were to 0.09 mÅ and 0.1 ms, respectively. The stimulating electrode was placed two ends of the regenerated nerve with five millimeter distance, while the recording electrode was placed proximal and distal to the gastrocnemius muscle. Subsequently, the compound muscle action potential (CMAP) latency and the peak amplitude were calculated separately.

#### CatWalk Gait Analysis

The motor function recovery 4, 8, and 12 weeks after surgery was evaluated using the CatWalk XT 9.0 gait analysis system (Noldus, Wageningen, Netherlands). Training was performed before the gait test to allow the rats to familiarize themselves on the runway environment. Sciatic functional index (SFI), which was first proposed by Bain et al. (1989), is a quantitative measure of the sciatic nerve function and calculated as follows: SFI = 109.5 (ETS – NTS)/NTS – 38.3 (EPL – NPL)/NPL + 13.3 (EIT – NIT)/NIT – 8.8. The formula is determined by the indicators of the footprint of the animal during walking.

#### Muscle Weight and Muscle Fiber Remodeling

At 12 weeks postoperation, the gastrocnemius muscle of the operative, and contralateral hind limbs was isolated and removed. After the muscle specimens were weighed immediately, they were fixed in 4% paraformaldehyde and then embedded with paraffin. The middle section of the muscle was cut into 7 mm thick sections and subjected to Masson’s trichrome staining. Eight random images from each sample were quantitatively analyzed using Image Pro Plus.

#### Statistical Analysis

Data were analyzed with SPSS 17 through one-way ANOVA followed by Tukey’s *post hoc* multiple comparison test. Results were presented as mean ± SD. *p* < 0.05 was considered as a statistically significant threshold of differences.

## Results

### Effect of SB216763 on DRG

**FIGURE 1:**
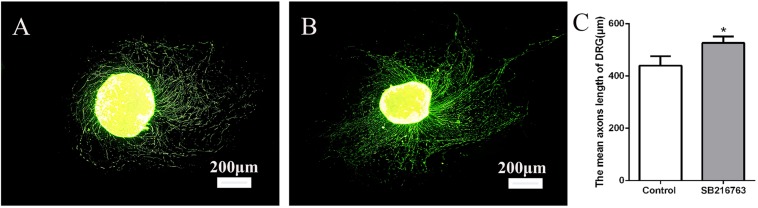
Effects of SB216763 on DRG. **(A)** NF-200 Immunofluorescence staining of DRG in the control group. **(B)** NF-200 immunofluorescence staining of DRG in the experimental group. **(C)** Statistics of the DRG length of the two groups. *p < 0.05.

The DRGs were cultured in media with 10 μmol SB216763 to evaluate the potential effect of SB216763 on nerve regeneration. The length of the DRG axons was markedly promoted by SB216763 compared with that of the control group (Figure 1), clearly indicating that SB216763 promoted DRG neurite outgrowth.

### Morphological Characterization

**FIGURE 2:**
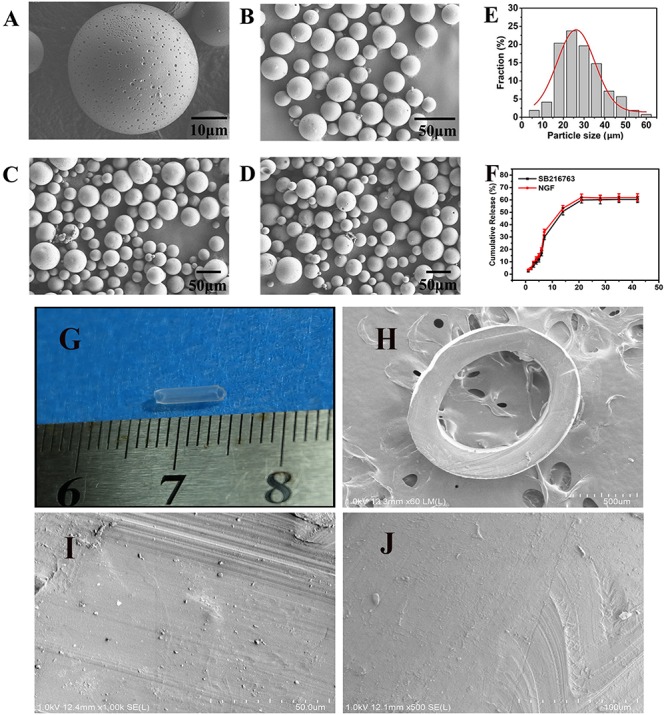
SEM images of **(A)** single microsphere and **(B–D)** three randomly selected locations. **(E)** Particle size distribution of microspheres and Gauss fitting. **(F)** *In vitro* release of SB216763 from PLGA microspheres at 37°C. **(G)** gross view of chitosan conduit. **(H)** SEM images of chitosan conduit transverse section. **(I)** SEM images of inner surface morphology of chitosan conduit. **(J)** SEM images of outer surface morphology of chitosan conduit.

SEM image in Figure 2A, the resulting PLGA microsphere displayed spherical shapes with some small sags. All of the spheres were complete (Figures 2B–D). The mean size of the microspheres was 28.34 μm after they were statistically evaluated and after all of the particle sizes of the microspheres were calculated. The particle size was essentially consistent with Gaussian distribution (*R*^2^ = 0.0928) as shown in Figure 2E. Figure 2G showed gross view of chitosan conduit. Figure 2H presented SEM images of chitosan conduit transverse section. Figure 2I represented SEM images of inner surface morphology of chitosan conduit. Figure 2J showed SEM images of outer surface morphology of chitosan conduit.

**FIGURE 3:**
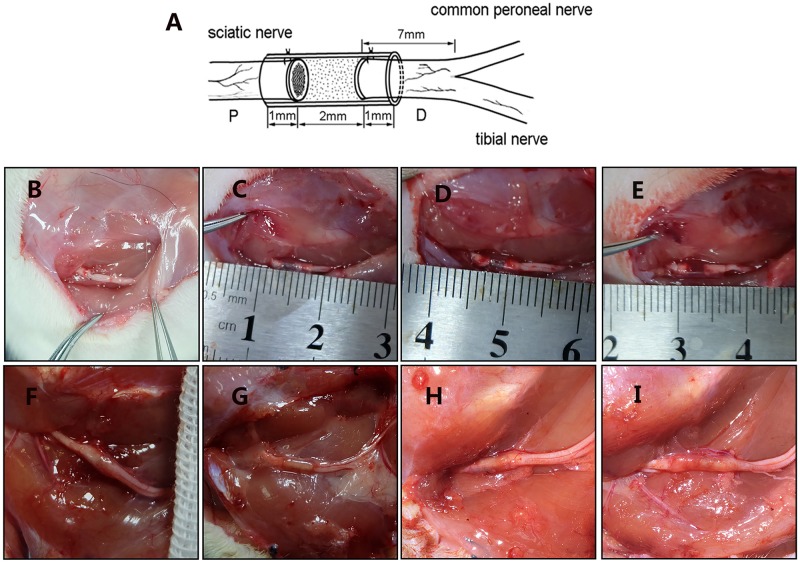
Surgical models and gross morphology 12 weeks postsurgery. **(A)** Schematic diagram of animal model. Figure is quoted from Wang et al. (2014). **(B)** Epineurium neurorrhaphy *in situ*. **(C)** Conduit small gap tubulization-integrated empty microspheres. **(D)** Conduit small gap tubulization-integrated SB-216763-loaded microspheres. **(E)** Conduit small gap tubulization-integrated NGF-loaded microspheres. **(F)** Gross morphology of epineurium neurorrhaphy. **(G)** Gross morphology of conduit small gap tubulization-integrated SB-216763-loaded microspheres. **(H)** Gross morphology of conduit small gap tubulization-integrated SB-216763-loaded microspheres. **(I)** Gross morphology of conduit small gap tubulization-integrated NGF-loaded microspheres.

#### *In vitro* Release Characteristics

The loading (w/w) and encapsulation (w/w) efficiency were 2.143 and 12.95% achieved for SB216763, 0.0022, and 12.89% achieved for NGF, respectively. In Figure 2F, on the first week, SB216763 had a burst release, and the microspheres released approximately 30% of the loading drugs. Then, the drugs were released slowly, and the cumulative release reached 50.7% at the end of the second week. The release rate continued to slow down during the third week, and the cumulative release was approximately 59.8%. The release of the drugs stopped in the fourth week, and the final cumulative release of SB216763 was approximately 60.4% of the total. The release tendency of NGF microspheres is similar to that of SB216763 microspheres. The final cumulative release was approximately 61.9%.

#### Morphological Analysis of the Regenerated Sciatic Nerve

**FIGURE 4:**
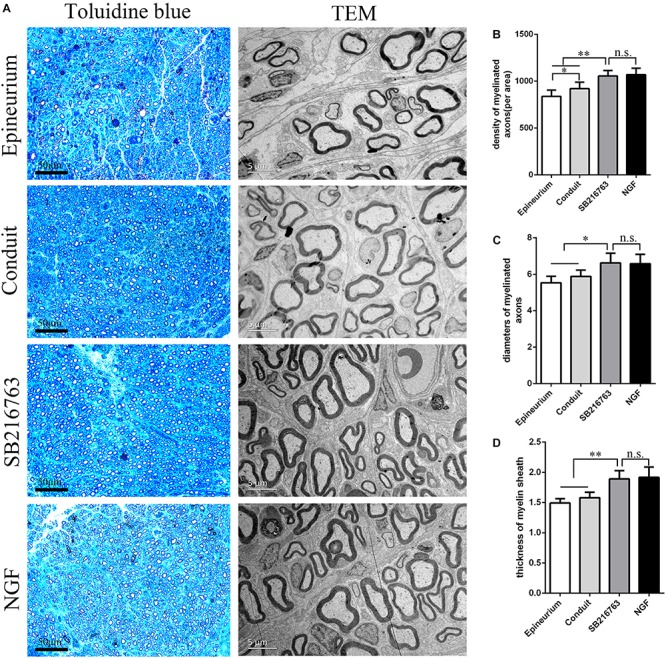
Evaluation of the regenerated nerve fibers 12 weeks after surgery. **(A)** Toluidine blue staining and TEM images of the regenerated sciatic nerve transverse sections. **(B)** Density of the myelinated nerve fibers. **(C)** Diameters of the myelinated nerve fibers. **(D)** Myelin sheat thickness. *p < 0.05 and **p < 0.01.

Figure 4A shows the regenerated nerve fibers isolated from the distal of the implants 12 weeks after surgery. The toluidine blue staining of the regenerated tissue revealed the number of the regenerated nerve fibers. The mean diameter and the myelin sheath thickness of the myelinated nerve fibers were quantified through TEM. Statistical analysis confirmed that the SB216763 group had more regenerated nerve fibers than the control group (Figure 4B), and the nerve fiber diameter and the myelin sheath thickness were larger than those of the control group (Figures 4C,D). However, the SB216763 group has no statistical difference with NGF group.

#### Electrophysiological Assessment

**FIGURE 5:**
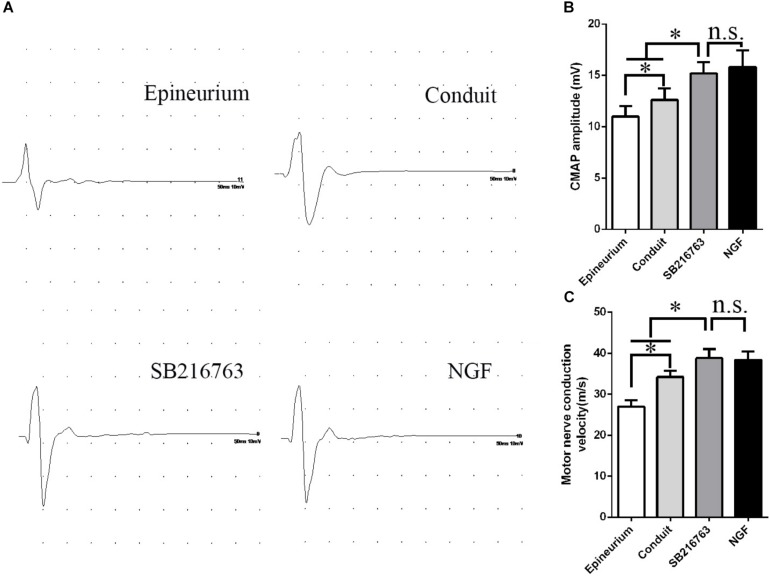
Electrophysiological examinations conducted 12 weeks after surgery. **(A)** Typical CMAP waveform at the operation side in each group. **(B)** Statistical analysis of CMAP amplitude. **(C)** Statistical analysis of NCV. *p < 0.05.

Electrophysiological examination was carried out 12 weeks after implantation (Figure 5). Figure 5A shows the representative latency and the amplitude of CMAP in all of the groups. The CMAP amplitude was determined by the number of the innervated muscle fibers, while CMAP latency depended on the degree of the remyelination of axons. The CMAP amplitude and nerve conduction velocity (NCV) values were calculated

The CMAP amplitude of the SB216763 group was higher than those of the two other groups, thereby indicating that the recovery of the muscle fibers was better than those of the two other groups (Figure 5B). The NCV of SB216763 group was faster

than those of the two other groups, thereby revealing that the remyelination of the axons of the regenerated nerve was better than those of the two other groups (Figure 5C). Also, the CMAP amplitude and NCV values of SB216763 group has no statistical difference with NGF group.

#### CatWalk Gait Analysis

**FIGURE 6:**
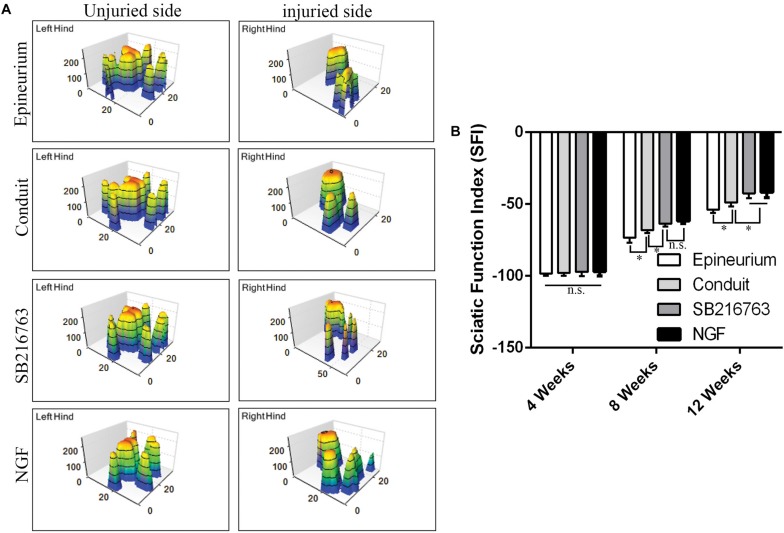
Motor function 12 weeks after surgery detected by CatWalk gait analysis. **(A)** Typical 3D plantar pressure distribution of the left and right hinds from all of the groups. **(B)** Statistical analysis of the SFI values of all of the groups. *p < 0.05.

The motor functional recovery 12 weeks after implantation was examined by the CatWalk gait analysis system (Figure 6). The stress area of the injured side of the SB216763 group was larger than that of the control group and similar to that of the NGF group (Figure 6A). SFI is a common method to evaluate the functional recovery of the injured sciatic nerves in rats. We found insignificant differences among the groups 4 weeks after surgery. The recovery of the SFI in the SB216763 group was better than that in the control group at 8 and 12 weeks after operation (Figure 6B).

#### Muscle Morphological Analysis

**FIGURE 7:**
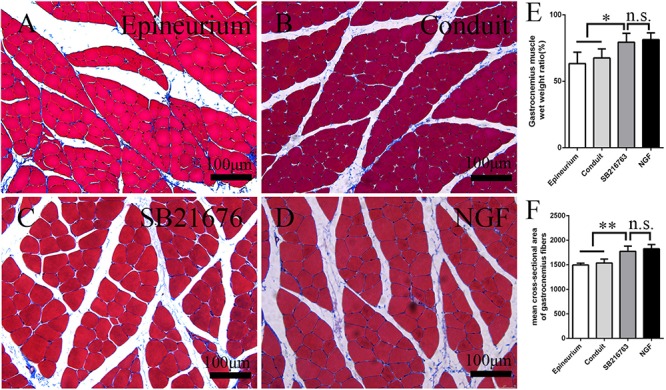
Evaluation of the gastrocnemius muscle 12 weeks after operation. **(A–D)** Masson’s trichrome staining images of the middle transverse sections of the gastrocnemius muscles. **(E)** Statistical analysis of the gastrocnemius muscle wet weight ratio. **(F)** Statistical analysis of the cross-section of the gastrocnemius muscle fiber. *p < 0.05 and **p < 0.01.

After peripheral nerve injury occurred, the gastrocnemius muscle began to undergo atrophy. The gastrocnemius muscle recovered gradually after surgery. It was isolated 12 weeks after operation, and histological examination was performed on the middle cross-section of the gastrocnemius muscle. The muscle fiber cross-sections of all of the groups were observed under an optical microscope (Figures 7A–D). The mean cross-sectional area of the muscle fiber of the SB216763 group was larger than that of the control group and (Figure 7F), and the muscle wet weight ratio was better than that of the control group (Figure 7E). This finding was consistent with the SFI results.

## Discussion

Small gap tubulization was performed on the basis of nerve-selective regeneration theory introduced by Ramóny Cajal et al. (1991). Conduit small gap tubulization is an important technological innovation. Our team has performed a considerable amount of animal experiments to confirm that conduit small gap tubulization is better than epineurium neurorrhaphy. We also found that 2 mm is the most suitable gap in the biological degradable conduit in rats. A clinical multicenter trial revealed that the recovery of the patients in the conduit small gap tubulization group was better than those in the epineurium neurorrhaphy group. Conduit small gap tubulization could reduce not only the operation time but also the occurrence of painful neuroma compared with traditional epineurium neurorrhaphy (Zhang et al., 2015b; Peixun et al., 2017).

The 2 mm gap allows fiber selective regeneration, thereby preventing the escape of the ruptured regenerating fibers. The chamber constructed with a 2 mm gap provides a microenvironment for peripheral nerve regeneration by adding various neurotrophic factors. In a previous study, our team added NGF to the small gap and found that the repair effect is better than that on the control group (Wang et al., 2014). However, the short half-life of neurotrophic factors limits their use. In this study, we used a small molecular compound, namely, SB216763, which is a GSK3β inhibitor, for sciatic nerve repair in rats.

Glycogen synthase kinase-3β has a wide range of cellular functions, including cell death, cell cycle, carcinogenesis, and autophagy, and it is considered an important regulator of various signal transduction pathways, including the Wnt pathway (Bhat et al., 2000; Forde and Dale, 2007; Park et al., 2013). GSK3β has also become an important drug target in anticancer therapy (Kingwell, 2018), Obesity-Induced White Adipose Tissue Inflammation (Wang et al., 2018), diabetes (Mussmann et al., 2007), and central nervous system diseases (Wang et al., 2017; Wickens et al., 2017).

However, few studies on GSK3β in the peripheral nervous system have been performed. Makoukji et al. (2012) used a nerve crush model to study the effect of GSK3β inhibitor on the remyelination of peripheral nerves. They found that the 00 inhibitor can increase the amount of myelinated nerve fibers and the thickness of myelin sheath. LiCl activates the Wnt signaling/β-catenin pathway, which is the basis of myelin gene expression. However, they used oral or intraperitoneal administration, which decreases the efficiency of the GSK3β inhibitor. SB216763 is a GSK3β-specific inhibitor and shows potential for treatments. Therefore, we combined conduit small gap tubulization with SB216763-loaded microspheres to repair peripheral nerve injury locally. In addition, sustained release microspheres can be used as drug delivery vehicles to maintain drug concentration and improve drug efficiency.

In this study, the base material of the microspheres was PLGA, which is a biodegradable material that has been widely used in polymeric microspheres, and the ratio of lactic acid to glycolic acid can be modulated to alter the release characteristics.

The result of the blank microsphere group was better than that of the control group, demonstrating that the PLGA microsphere delivery system did not inhibit or obstruct the outgrowth of nerve fibers. This finding is consistent with our previous research (Wang et al., 2014).

Most studies on drug delivery have been performed to repair nerve crush injury (Li et al., 2017a, b, 2018). Li et al. (2018) developed a thermo-sensitive heparin-poloxamer (HP) hydrogel co-delivered with basic fibroblast growth factor and NGF in diabetic rats with sciatic nerve crush injury. However, our study aimed to enhance the repair effect on nerve transection injury.

*In vitro* experiments confirmed that approximately 60% of the encapsulated SB216763 was released from the SB216763-loaded microspheres. The concentration of the SB216763 solution was set to 10 μM (Makoukji et al., 2012). *In vitro*, we used the tissue culture method and found that SB216763 could promote DRG neurite growth for the first time. Our previous study also confirmed that SB216763 could upregulate myelin proteins (MPZ and PMP22) and remyelination transcription factors (Oct6 and Sox10) (Chen et al., 2016).

*In vivo*, histological evaluation indicated that the number of myelinated nerve fibers and the thickness of myelin sheaths in the SB216763 group were superior to those in the control group.

This result could be attributed to the capability of SB216763 to promote nerve growth and the continuous formation of myelinated nerve fibers the 2 mm microenvironment.

Electrophysiological assessment showed that the CMAP latency in the SB216763 group was lower than that in the control group, whereas the CMAP amplitude in the SB216763 group was higher than that in the control group. These results revealed indirectly that the myelinated nerve fibers and the myelin sheath thickness in the SB216763 group were greater than those in the control group.

The recovery of muscle function and sciatic nerve index in the SB216763 group was superior to the control group, further confirming the role of the local application of SB216763 in the small gap after peripheral nerve injury.

In brief, the use of conduit small gap tubulization-integrated small molecule compound-loaded microspheres in our research is essential for providing patients with a new treatment method for peripheral nerve injury in clinics without the limitations of high cost, short half-life, and controversial sources.

## Ethics Statement

We followed the Laboratory Animal Guideline for the Ethical Review of the Animal Welfare of China (GB/T 35892-2018). All of the experiments were in compliance with the relevant regulations of the Medical Ethics Committee of Peking University People’s Hospital (Approval No. 2102000024).

## Author Contributions

FR, ZY, and DZ performed the majority of the experiments, analyzed the data, and prepared the manuscript. FY helped with the cell culture. ML and DL helped with *in vivo* experiments. BJ, YW, and PZ supervised the project and wrote most of the manuscript.

## Conflict of Interest Statement

The authors declare that the research was conducted in the absence of any commercial or financial relationships that could be construed as a potential conflict of interest.
